# Comparison of three methods for mitochondria isolation from the human liver cell line *(HepG2)*

**Published:** 2016

**Authors:** Pedram Azimzadeh, Hamid Asadzadeh Aghdaei, Peyman Tarban, Mohammad Mahdi Akhondi, Abolfazl Shirazi, Hamid Reza Khorram Khorshid

**Affiliations:** 1 *Reproductive Biotechnology Research Center, Avicenna Research Institute, ACECR, Tehran, Iran *; 2 *Basic and Molecular Epidemiology of Gastrointestinal Disorders, Research Institute for Gastroenterology and Liver Diseases, Shahid Beheshti University of Medical Sciences, Tehran, Iran*; 3 *Genetic Research Center, University of Social Welfare and Rehabilitation Sciences, Tehran, Iran*

**Keywords:** Mitochondrial isolation, Cell line, *HepG2*

## Abstract

**Aim:**

The aim of this study was to evaluate and compare three available methods for mitochondrial isolation from a human cell line to predict the best method for each probable application.

**Background:**

Organelle isolation is gaining importance in experimental laboratory settings. Mitochondrial dysfunction may affect tumorgenesis process. There are some evidences that transplantation of healthy, intact and active mitochondria into cells containing defective mitochondria may reduce the proliferation. Therefore, isolated mitochondria could be considered as an effective tool for assessment and management of mitochondrial related disorders.

**Patients and methods:**

Mitochondrial isolation from the human liver cell line (*HepG2*) was performed using two commercially available kits, including Qproteome (Qiagen) and MITOISO2 (Sigma-Aldrich), as well as a manual method. Integrity of inner membrane of mitochondria was assessed by JC-1 staining. Activity of isolated mitochondria was evaluated by DCFH-DA staining, and total yield of isolated mitochondria determined by micro-Lowry method. Finally, relative quantification using Real-time PCR was conducted to compare the mtDNA copy number of mitochondria isolated by three different methods.

**Results:**

Compared to other methods, manual kit resulted in higher yields of total amount of mitochondrial protein and mtDNA copy numbers. Isolated mitochondria by Qproteome kit, showed a higher activity. Finally, the integrity of inner-membrane of isolated mitochondria was significantly higher in Qproteome when compared with the other two methods.

**Conclusion:**

Due to differences in quality, quantity and activity of isolated mitochondria using three techniques discussed here, the method in which best-suited to each research project should be selected according to the distinct features of isolated mitochondria.

## Introduction

 Organelle analysis is a powerful strategy to discover the pathophysiology of human diseases (1, 2). There are established methods for isolation of lysosomes, peroxisomes, endoplasmic reticulum and mitochondria (3-5). One of the most useful applications of the organelle isolation is to determine the localization of proteins within cells (6). Proteomics analysis of mitochondria also needs pure and intact isolated mitochondria in the first step (7). Mitochondrial proteome consists of 13 proteins encoded by mtDNA, which are involved in cellular respiration. However, the rest of mitochondrial proteins are encoded by nuclear genome, synthesized in cytosolic ribosomes and imported into the organelle (8). It has been shown that the number of mitochondria varies among different cells (9, 10). Therefore, design and application of suitable mitochondria isolation methods for different types of cells with different amounts of mitochondria could be helpful (11, 12). 

There are two main reasons for biomedical scientists to study about mitochondria, including: discovery of the genetic basis of several mitochondrial diseases and targeting mitochondria for gene therapy. The next step in this process would be gene replacement to prevent from mitochondrial disorders (13). The second reason to study mitochondria is its main role in the production of reactive oxygen species (ROS) (14). ROS production induces mitochondrial dysfunction, apoptosis and necrosis while cells undergoing aging course. ROS production is also integrated with mitochondrial redox signaling (15). Accordingly, the amount of ROS produced by mitochondria is a marker for the determination of mitochondrial activity (15). ROS production can be detected using Dichloro-dihydro-fluorescein diacetate (DCFH-DA) dye. DCFH-DA is a non-polar, non-fluorescent dye that turns to a highly fluorescent derivative of DCF when oxidized by H_2_O_2_ or other ROS (16). 

The quantity of isolated mitochondria could be determined by assessment of total protein content and mtDNA copy number. Two main methods are available for measurement of total protein content. Biuret and Lowry procedures, former is more prevalent in clinical tests and the latter is convenient for analytical works because of its higher sensitivity (17). 

Preservation of membrane integrity is another important factor during mitochondria isolation. TMRM, Rhod123, JC-1 and DiOC_6 _are typical probes for measurement of mitochondrial membrane potential. JC-1 is a widely used dye for measurement of inner-membrane potential of isolated mitochondria (18).

Some evidences show the transplantation of healthy, intact and active mitochondria into cells containing defective ones could be used as a potential healing agent for a variety of diseases (19). Therefore, we need a reliable source of mitochondria for isolation and available source of recipient cells. For further genetics or biochemical analysis, human cell lines are considered as a good source for mitochondria isolation, due to their easy identification, cultivation and availability (20). 

The aim of this study was to compare three available methods for mitochondrial isolation with regard to their suitability for different requirements on isolated mitochondria. 

## Patients and Methods

**Cell Culture**

Human liver cell line *HepG2* was obtained from National Cell Bank (*Pasteur* Institute of Iran). *HepG2 *cells were cultured in 25 cm^2^ tissue culture flasks (SPL, China) containing RPMI 1640 media (Gibco, USA), supplemented with 10% heat inactivated Fetal Bovine Serum (FBS) (Gibco, USA), and 1% (v/v) penicillin-streptomycin (Sigma-Aldrich, USA). Adherent cells were detached using trypsin-EDTA solution (Gibco, USA), containing 0.05 % trypsin and 0.5 mM EDTA, at 80% confluence. Cells were counted and centrifuged at 2000 rpm (R: 10.70). 

**Isolation of Mitochondria**

Mitochondrial isolation was performed using three available methods, including two commercial kits and one manual method. Qproteum mitochondria isolation kit (Qiagen, USA), and MITOISO2 mitochondria isolation kit (Sigma, USA) were commercial kits. The manual isolation method was performed using differential centrifugation as previously described by Dhruv Sareen, *et al*. (21) with some modifications. Isolation basis for two selected kits are quite related, but including them in such analyzes may help to elucidate the best way to use them. 

Briefly, we used the same extraction buffer, but in the homogenization step dounce homogenizer was used to have a gentler treatment with mitochondria exerted from cells. Another difference was in the buffer in which activity assay was performed. We have found a respiratory buffer described by Mashayekhi *et al*. (22) more practical with more clear results. MOPS, MgCl_2_, Sodium succinate and EGTA were commonly found in two buffers. However, the respiratory buffer includes KH_2_PO_4_ instead of KCl. 

**Quantity of Isolated Mitochondria**

Total protein content has been considered as a marker for quantity of isolated mitochondria (4). To assess the micrograms of isolated mitochondrial protein, there were two major choices, including: Biuret and Lowry methods. Biuret is more suitable for high concentrations of mitochondria and the Lowry method instead recommended due to its high sensitivity (23). The micro-Lowry method with Onishi and Barr Modification (TP0200, Sigma-Aldrich, USA) was used according to manufacturer’s instructions. Briefly, this method is based on two main reactions, including: biuret reaction and a reduction reaction that yields a purple color. Finally, reading the absorbance of the colored solution at 500 nm and 800 nm provided a raw data. Then, a standard curve delineated and micrograms of unknown samples were determined. To draw the standard curve, a 400-µg/mL stock was diluted in water into 50, 100, 200 and 300 µg/mL aliquots. After determining the protein concentration of each sample, results were multiplied by the dilution factor to obtain the total protein concentration of isolated mitochondria using each method. 

Relative mtDNA copy numbers of isolated mitochondria by three methods were also measured using a SYBR green based quantitative real-time PCR assay as described before (24). Essential primers for detection and amplification of mtDNA were designed for tRNA^Leu (UUR)^ gene (Table-1). All tests were repeated three times, conducted in triplicate and β-2-microglobulin was used as an endogenous control for normalization of relative quantification test results (24). The ROX fluorescent dye was added to the real-time PCR master mix as a background dye to normalize the fluorescent signals in each reaction (25). 

DNA was extracted QIAamp using DNA mini kit (Qiagen, USA) with some modifications. Briefly, mtDNA was extracted according to manufacturer’s instructions; however, RNase A treatment was eliminated. This is due to the negative effect of RNase treatment on mtDNA that is mainly a DNA-RNA hybrid. RNase treatment probably is the way that ribonucleotides are in contact with close circular mtDNA and consequently lower the rate of mtDNA yield (26-29). All PCR procedures were performed using RNase Free material and plastic-ware.

Quantitative PCR mix (Power SYBR® Green PCR Master Mix) was obtained from ABI (Life technologies, USA) and primers were synthesized by Macrogen Co. (Korea) with additional High-Performance Liquid Chromatography (HPLC) purification. 

**Table 1:** Sequence of primers used for quantitative Real time PCR

Primer Name	Sequence	Amplicon Size
β-2-microglobulin	Forward: TGCTGTCTCCATGTTTGATGTATCT Reverse: TCTCTGCTCCCCACCTCTAAGT	86 bp
tRNALeu ^(UUR)^	Forward: CACCCAAGAACAGGGTTTGT Reverse: TGGCCATGGGTATGTTGTTA	107 bp

Relative quantification was performed using ABI7500 Real-time PCR system (Applied Biosystems, USA). To confirm the quality and efficiency of PCR amplification, a dissociation step (Melt curve analysis) after regular amplification cycles with six serial dilutions of extracted mtDNA was performed. High quality and efficient amplification should demonstrate at least one peak in melt curve analysis and the slope of standard curve was used to calculate the efficiency as previously described (30).

The relative mtDNA copy number was calculated using the Delta C_T_ method (24). The C_T _value of each sample, which obtained from amplification of tRNA^Leu(UUR)^ gene was normalized with a C_T _value of β-2-microglobulin to create the Delta C_T_. To calculate the Delta C_T_ value and relative mtDNA content, each method was considered as calibrator once and compared the amounts of mtDNA yielded from two other methods with the calibrator. 

**Quality and Membrane Integrity of Isolated Mitochondria**

Quality of isolated mitochondria and the integrity of membrane were assessed using the JC-1 test by Mitochondria Staining Kit (CS0390, Sigma, USA). This test is based on electrochemical proton gradient of mitochondrial inner membrane (18, 31). 

**Activity of Isolated Mitochondria**

ROS production was measured using 2’, 7’-Dichloro-dihydro-fluorescein diacetate (DCFH-DA) (Sigma, USA) assay. This could be considered as a marker for mitochondrial activity due to production of hydrogen peroxide (H_2_O_2_) and some other ROS. DCFH-DA produces green fluorescence in the presence of ROS (32). As mentioned before, the respiratory buffer was used as a reaction medium to assay the amount of ROS released from mitochondria. 

**Figure 1:**
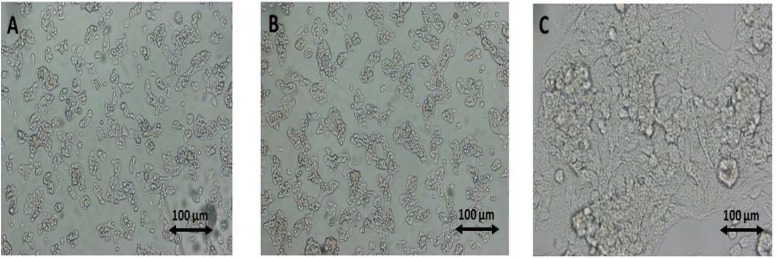
Sequential *HepG2* cell culture captures at day-1 (A), day-3 (B) and day-6 (C).

Briefly, we added DCFH-DA to the isolated mitochondria (0.4 µg of protein) in a final concentration of 10 µM and incubated for 15 minutes at room temperature. Emission of fluorescent was determined using the BD FACS calibure (Becton Dickinson, Anaheim, CA), and flow cytometer. Fluorescent intensity was determined for 10000 counts. 

**Statistical Analysis**

Comparison of quantitative variables such as total protein content, fluorescent emission of DCFH-DA dye and JC-1 dye between three groups of samples (Isolated using three different methods) were conducted using one-way ANOVA (Kruskal-Wallis) test. Data were analyzed and plotted using ABI7500 software ver.2.0.1 (Life technologies, USA) and GraphPad Prism ver. 5.0 (GraphPad Software Inc., La Jolla, CA). 

## Results

**Figure 2:**
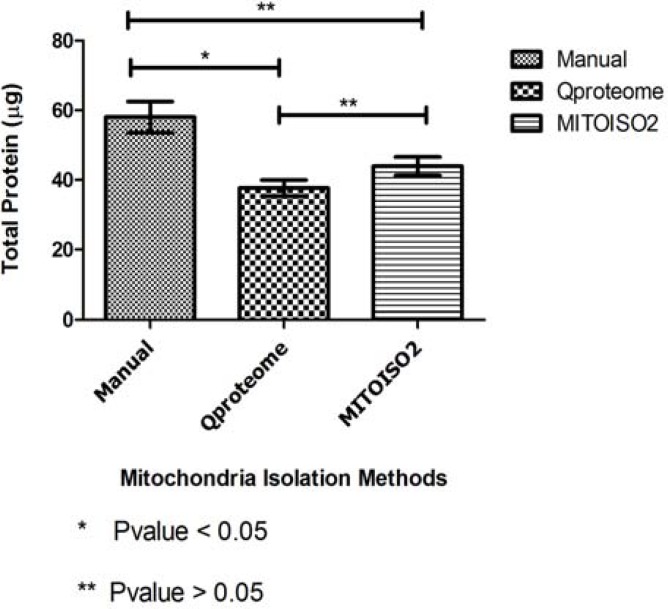
Comparison of total protein yield among isolated mitochondria with three methods

Cultured *HepG2* cells were confluent in day 1, 3 and 6 (Figure 1). Mitochondrial isolation was conducted by three methods on triplicate reactions. Measurement of total protein content of isolated mitochondria (from 10^6^ cultured cells) using micro-Lowry method revealed that manual differential centrifugation method provides more mitochondria (58.03 µg) than the MITOISO2 kit (43.9 µg). Among these methods, Qproteum kit (37.6 µg) had the lowest yield (Figure 2). The difference between the total protein content of Qproteome kit and manual method was statistically significant (P= 0.032). However, no significant difference was observed between MITOISO2 and Qproteome kits.

**Figure 3:**
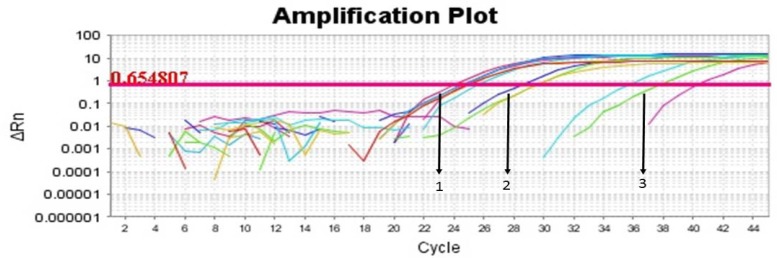
mtDNA copy number amplification plot. Curves (1) represent mtDNA amplification of samples isolated with Manual method. Curves 1 and 3 represent mtDNA amplification of samples from MITOISO2 and Qproteome kits, respectively

Relative mtDNA copy number calculation revealed that mtDNA content of manual method was 3.4 fold and 2.2 fold higher than Qproteome and MITOISO2 kits, respectively (Figure 3). These findings confirmed results of protein assay. 

**Figure 4:**
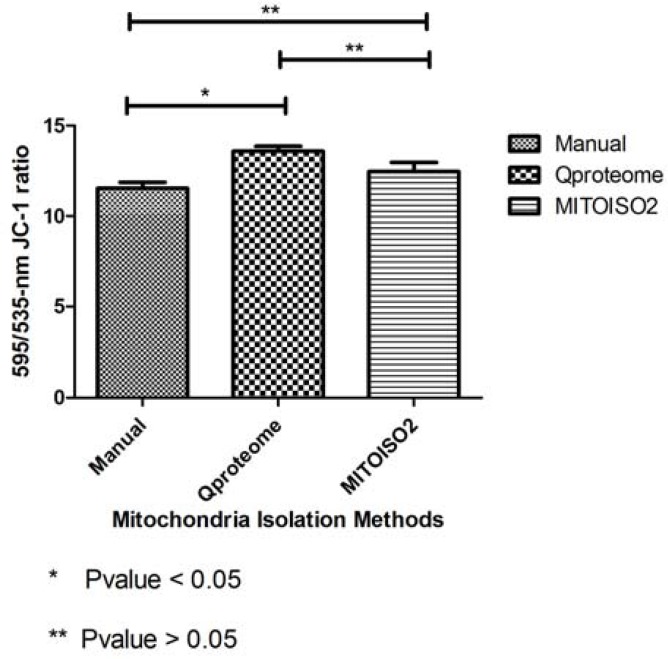
Comparison of membrane integrity of isolated mitochondria with three methods

Comparison of mitochondrial membrane integrity was performed using JC-1 staining and showed Qproteome kit provides more intact mitochondria and the isolated mitochondria by manual method had the lowest rank of membrane integrity (Figure 4). The difference between Qproteome kit and manual method indicated as an absorbance ratio of 595/535 nm JC-1 was statistically significant (P= 0.032). However, MITOISO2 and Qproteome kits showed no significant difference (P>0.05). 

**Figure 5:**
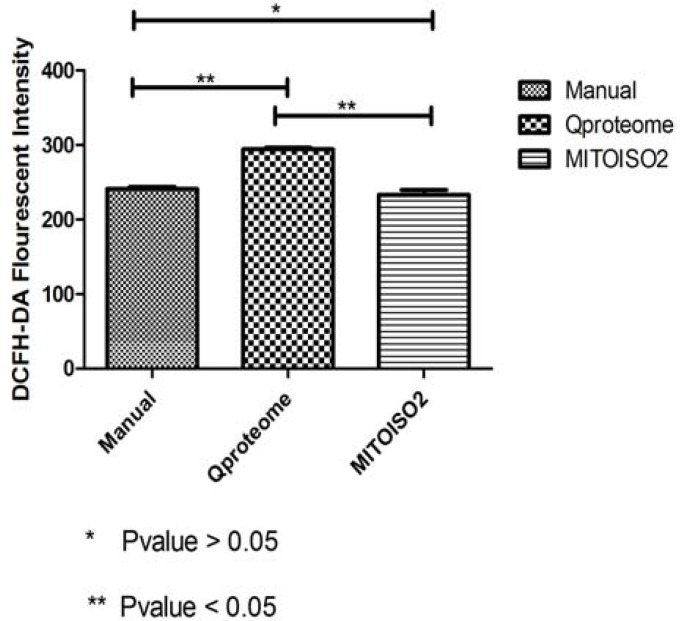
Comparison of ROS production of isolated mitochondria with three methods

Results of mitochondrial activity assays (Defined as fluorescent intensity per 10^6^ cells) showed that mitochondria isolated using the manual method and the MITOISO2 kit have quite similar activities (P>0.05). Mitochondria isolated with Qproteome showed a statistically significant high fluorescent intensity when compared to other methods (P= 0.002) (Figure 5). 

## Discussion

Despite many available procedures for *in-vitro* analysis of mitochondria, investigating intact and active isolated mitochondria from different cell types offer more unmatched applications. So far, many researchers have been used manual and kit-based organelle isolation methods. In the present study, the attributes of isolated mitochondria by three common methods were compared to answer this question. We have found that manual mitochondrial isolation method yields more mtDNA copy numbers in comparison with two kit-based methods. This is in consistence with the results of total protein determination using micro-Lowry method. However, the inner-membrane potential assay and mitochondrial activity assays indicated that the proportion of intact and active mitochondria in kit-based methods is more than manual method. 

To the best of our knowledge, there are limited studies regarding the comparison of different methods for mitochondrial isolation. Hartwig *et al*. in 2011, published a technical brief on classical and kit based methods for isolation of mitochondria from mouse liver and compared the total mitochondria yield, mitochondrial activity and purity (4). Accordingly, we did not find any articles that compared such methods for isolation of mitochondria from human liver tissue or cell lines. 

Since isolated mitochondria are targeted to transfer into new cells, several methods have been developed to transfer isolated mitochondria into live cells. Microinjection is the most prevalent method and biochemical methods such as peptide mediated mitochondrial delivery (PMD) and liposome mediated transfer are now available (33).

Sims *et al*. in 1990, developed a rapid isolation method of active mitochondria from rat brain and it’s sub-regions (5). This study introduced two protocols based on discontinuous percoll gradient, for mitochondrial isolation according to further applications. 

Pooreydy *et al*. offered a cost-effective and time-efficient method to isolate relatively pure mitochondria from PBMCs with focus on proteomics issues (34). The above-mentioned method seems to be cost effective and didn’t require special instruments such as ultracentrifuge. Due to difference in quality assays between this study and our investigation, we haven’t compared it with results of the present study. Several other studies have isolated the mitochondria from tissues and cells using manual methods consisted of sequential steps of homogenization, washing and centrifugation in 600g to 11000g (21, 22, 35). 

Added to these data, several studies have been used kit-based method for isolation of mitochondria especially for further injection strategies. Elliot *et al*. isolated mitochondria from MCF-12A human mammary gland cell line using MITOISO2 kit and transplanted the isolated mitochondria into live cells. Their results showed that introduced mitochondria are acceptably active (19). Qproteome kit was also applied previously for isolation of mitochondria for both organelle transplantation and proteomics analysis (36). 

Main differences among three studied methods for mitochondrial isolation were in time efficiency, cost, equipment needed, quality and quantity of isolated mitochondria. All three methods yielded a proportion of intact and active mitochondria. With regard to these criteria, one can choose what method best suits their work.

Isolated mitochondria could be considered as an effective tool for proteomics analysis, mtDNA scrutiny and cell based therapies. If a large amount of mitochondrial fractions for proteomics or such reasons is required, the manual method is the most suitable and cost effective method but if the purpose of isolation is to introduce the intact and active mitochondria into live cells using microinjection, biological or chemical methods, the Qproteome is the true choice. Although, mitochondria isolated by MITOISO2 have been shown quite similar inner-membrane integrity but is provides mitochondria with significantly lower activity according to amount of ROS production.

## Note

**(**Please cite as:** Azimzadeh** **P, Asadzadeh Aghdaei** **H, Tarban** **P, Akhondi** **MM, Shirazi** **A, Khorram Khorshid HR. Comparison of Three methods for mitochondria isolation from human liver cell line (*****HepG2*****). Gastroenterol Hepatol ****Bed Bench**** 2016;9(2):105-113).**
